# 180. Durability of Antibody Response to RSV Vaccination in Immunocompromised Individuals

**DOI:** 10.1093/ofid/ofaf695.059

**Published:** 2026-01-11

**Authors:** Zeba Nauroz, Camille Hage, Isabella Sengsouk, Prasanthy Balasubramanian, Xori Green, Woudase Gallo, Jiashu Xue, Maggie Chahoud, Aaron Tobian, William Werbel, Andrew H Karaba

**Affiliations:** Johns Hopkins, Baltimore, Maryland; Johns Hopkins University, Baltimore, Maryland; Johns Hopkins University, Baltimore, Maryland; Johns Hopkins, Baltimore, Maryland; Johns Hopkins University, Baltimore, Maryland; Johns Hopkins, Baltimore, Maryland; Johns Hopkins University, Baltimore, Maryland; Johns Hopkins University, Baltimore, Maryland; Johns Hopkins University, Baltimore, Maryland; Johns Hopkins University, Baltimore, Maryland; Johns Hopkins University, Baltimore, Maryland

## Abstract

**Background:**

Immunocompromised persons (ISPs) demonstrate attenuated antibody responses to respiratory syncytial virus (RSV) vaccination. In healthy populations, antibody (Ab) response and clinical protection last for >1 year following a single vaccine dose, yet Ab durability in ISPs has not been established.

**Figure:**
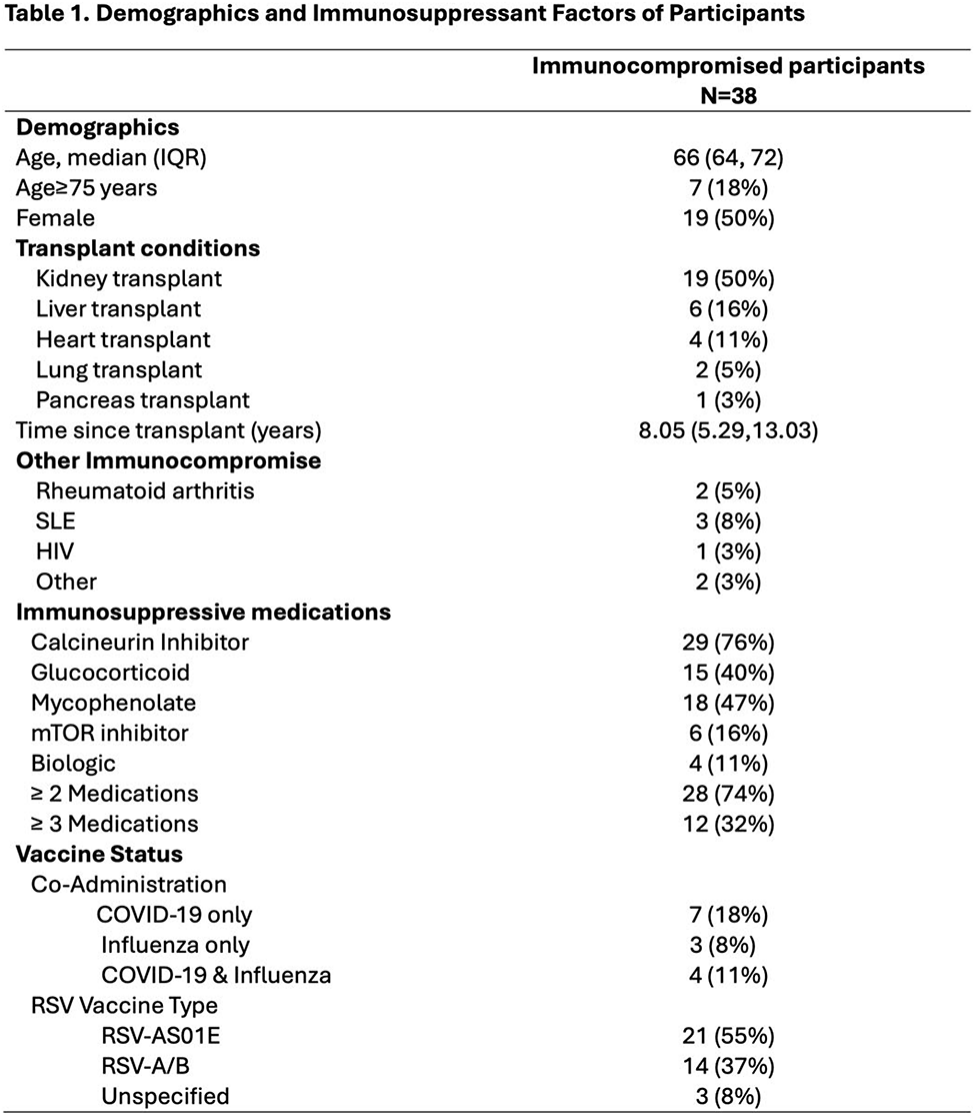

**Figure 1.**
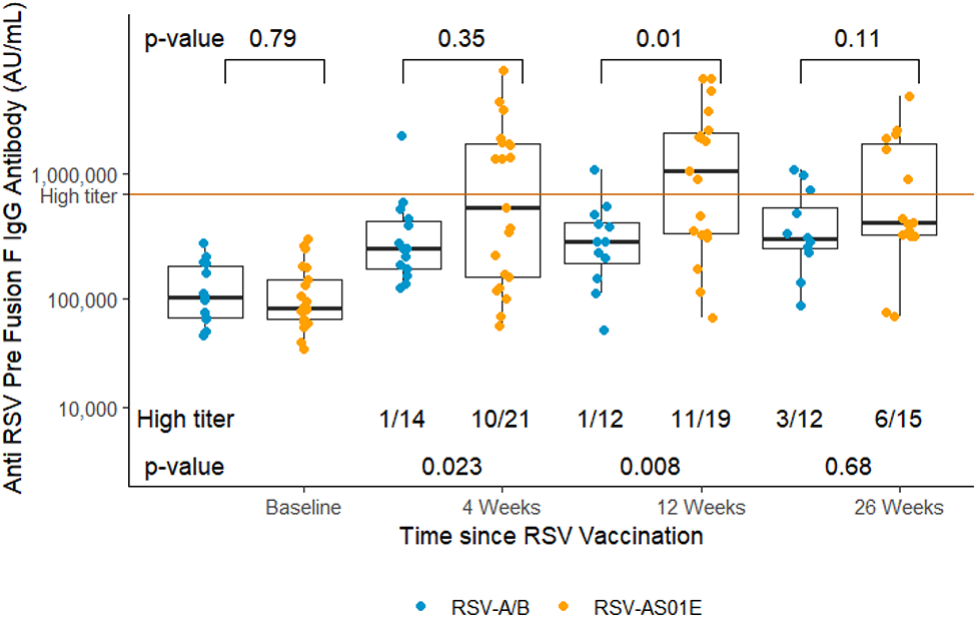
Anti-RSV preF IgG After Vaccination Over Time.

Total anti-RSV preF specific IgG (Arbitrary Units[AU]/mL) at baseline and the indicated number of weeks (W) after RSV vaccination. Each dot represents an individual sample. Dots are colored by vaccine type (Orange = RSV-AS01E, Blue = RSV A/B). Red horizontal line represents preF IgG value of high-titer pre-vaccine era control plasma from BEI resources. Fractions below the boxplots represent the participants from each vaccine type with high titer response over total recipients of that vaccine with available data at that time point. P values below the boxplot were computed by Fisher’s exact test. Brackets and p values comparing absolute antibody level between vaccine groups above the boxplots were computed using Wilcoxon rank-sum testing.

**Methods:**

In a national prospective cohort, we measured anti-RSV pre-fusion F IgG (PreF Ab) using an electrochemiluminescence assay (Meso Scale Discovery) and neutralizing titer using live-virus in ISPs who received the RSVPreF3 (GSK, AREXVY™, RSV-AS01E) or RSVpreF (Pfizer, ABRYSVO™, RSV-A/B) vaccines between October 2023 - October 2024 at baseline, 4, 12, and 26-weeks post-vaccination. Proportion achieving high-titer response (≥PreF Ab and NT50 of high-titer control plasma) were calculated and compared by vaccine type.

**Figure 2.**
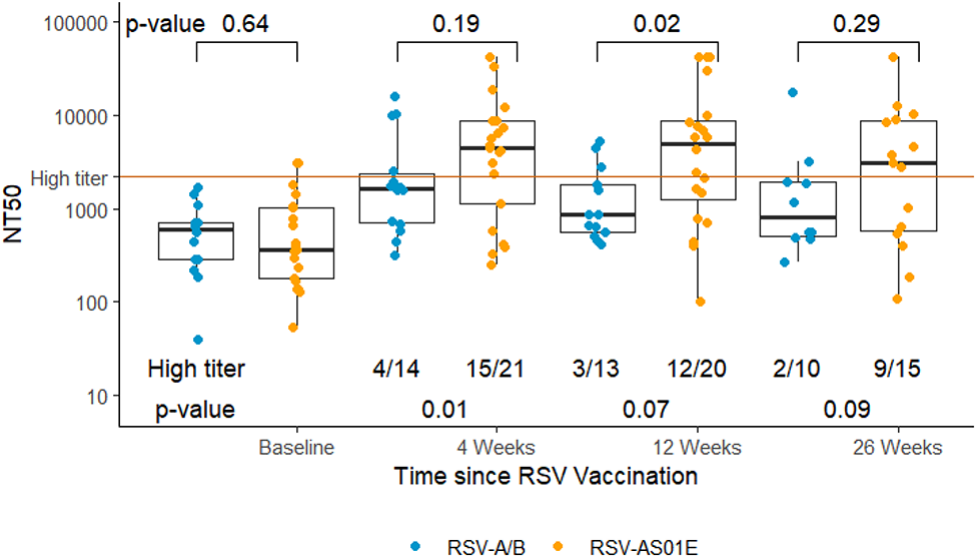
RSV Neutralizing Response After Vaccination Over Time.

Neutralizing titer 50 (NT50) at baseline and the indicated number of weeks (W) after RSV vaccination using plaque reduction assay with live RSV A2. Each dot represents an individual sample. Dots are colored by vaccine type (Orange = RSV-AS01E, Blue = RSV A/B). Red horizontal line represents NT50 value of high-titer pre-vaccine era control plasma from BEI resources. Fractions below the boxplots represent the participants from each vaccine type with high titer response over total recipients of that vaccine with available data at that time point. P values below the boxplot were computed by Fisher’s exact test. Brackets and p values comparing absolute antibody level between vaccine groups above the boxplots were computed using Wilcoxon rank-sum testing.

**Results:**

Among 38 vaccinated participants, median [IQR] PreF Ab rose from 87,292 AU/mL [64,088 – 185,854] to a peak of 529,608 AU/mL [ 320,400 - 2,636,592] at 12 weeks post-vaccine (p< 0.001 vs. baseline). At baseline, 4, 12, 26 weeks, high-titer PreF Ab response was present in 0/38 (0%),14/38 (37%), 13/33 (39%), and 11/30 (37%) participants, respectively. A higher proportion of RSV-AS01E vs. RSV-A/B recipients had high-titer PreF Ab and NT50 neutralization at each time point, with the greatest difference at 12 and 4 weeks respectively (11/19 vs. 1/12, p = 0.008 for PreF Ab and 16/23 vs 4/18 for NT50, p=0.004).

**Conclusion:**

Longitudinal data indicate relatively stable Ab levels out to 6 months post RSV vaccination in ISPs. However, many ISPs never achieve conservative thresholds of Ab response, suggesting ongoing vulnerability to infection and supporting investigation of additional primary vaccinations for this group.

**Disclosures:**

William Werbel, MD PhD, AstraZenca: Advisor/Consultant Andrew H. Karaba, MD PhD, GSK: Advisor/Consultant

